# Characterizing the Impacts of 2024 Total Solar Eclipse Using New York State Mesonet Data

**DOI:** 10.1029/2024GL112684

**Published:** 2024-11-20

**Authors:** Junhong Wang, Aiguo Dai, Chau‐Lam Yu, Bhupal Shrestha, D. J. McGuinnes, Nathan Bain

**Affiliations:** ^1^ New York State Mesonet SUNY University at Albany Albany NY USA; ^2^ Department of Atmospheric and Environmental Sciences SUNY University at Albany Albany NY USA

**Keywords:** total solar eclipse, New York State Mesonet, eclipse impacts, boundary layer, land‐atmosphere interactions

## Abstract

On 8 April 2024, a rare total solar eclipse (TSE) passed over western New York State (NYS), the first since 1925 and the last one until 2079. The NYS Mesonet (NYSM) consisting of 126 weather stations with 55 on the totality path provides unprecedented surface, profile, and flux data and camera images during the TSE. Here we use NYSM observations to characterize the TSE's impacts at the surface, in the planetary boundary layer (PBL), and on surface fluxes and CO_2_ concentrations. The TSE‐induced peak surface cooling occurs 17 min after the totality and is 2.8°C on average with a maximum of 6.8°C. It results in night‐like surface inversion, calm winds, and reduced vertical motion and mixing, leading to the shallowing of the PBL and its moistening. Surface sensible, latent and ground heat fluxes all decrease whereas near‐surface CO_2_ concentration rises as photosynthesis slows down.

## Key Points

- The New York State Mesonet provided unprecedented surface, profile, flux and image data during the 8 April 2024 total solar eclipse across New York State
- The eclipse resulted in significant cooling and moistening near the surface and in the boundary layer, leading to a surface inversion layer
- It also weakened surface winds, turbulent mixing, heat fluxes, but caused a robust rise in near‐surface CO_2_ concentrations

## 1 Introduction

Total solar eclipse (TSE) happens when the moon completely obscures the sun. TSEs are rare events and only last for a few minutes at any location. Each century there are about 50–70 TSEs around the globe (NASA, 2023). The contiguous U.S. has only three TSEs in the first half of this century, 2017, 2024 and 2045. The TSE on 8 April 2024 swept across North America from Sinaloa, Mexico through 15 U.S. states to Newfoundland, Canada. It lasted twice as long as that on 21 August 2017 and had more visible and larger sun's corona during totality because the sun was close to the solar maximum. The 2024 TSE was the only one that passes NYS since 24 January 1925 and would be the last one before 1 May 2079.

The effects of solar eclipses on Earth's atmosphere have been studied around the globe (e.g., Amiridis et al., 2007; Anderson et al., 1972; Aplin & Harrison, 2003; Eaton et al., 1997; Founda et al., 2007; Fowler et al., 2019; Hanna, 2000; Hanna et al., 2022; Harrison and Hanna, 2016; Mahmood et al., 2020; Pasken et al., 2023; Ramchandran et al., 2002; Spangrude et al., 2023; Szalowski, 2002). Surface temperature drops during the eclipses by 1–10°C during 40+ past eclipses depending on the time of the day, season, location, cloud cover, and other synoptic conditions (Aplin et al., 2016; Kameda et al., 2009). Changes in surface pressure, wind and humidity, and clouds have also been reported during solar eclipses (e.g., Anfossi et al., 2004; Aplin et al., 2016; Aplin & Harrison, 2003; G. Harrison and Gray, 2017; Trees et al., 2024). However, only a few studies used data from operational meteorological networks (e.g., Fowler et al., 2019; Hanna et al., 2022; Mahmood et al., 2020; Pasken et al., 2023).

Despite extensive documentation on surface meteorological responses to TSEs, much less effort has been devoted to study the responses of the planetary boundary layer (PBL). This is partially due to lack of upper air observations with sufficient temporal resolution to capture the short lifetime of an eclipse. Turner et al. (2018) and Mahmood et al. (2020) demonstrated the advantage of continuous PBL measurements using temporally deployed ground‐based remote sensing instruments at a limited number of sites to detect the collapse and reformation of the PBL during the 2017 TSE. Prior studies have also found significant weakening of surface energy fluxes during TSEs (e.g., Nymphas et al., 2012; Turner et al., 2018; Wood et al., 2019). However, to the best of our knowledge, *co‐located continuous surface, vertical profile, and surface flux measurements around and during the TSEs* had not been made before on a mesoscale network until the 2024 TSE.

**Figure 1:**
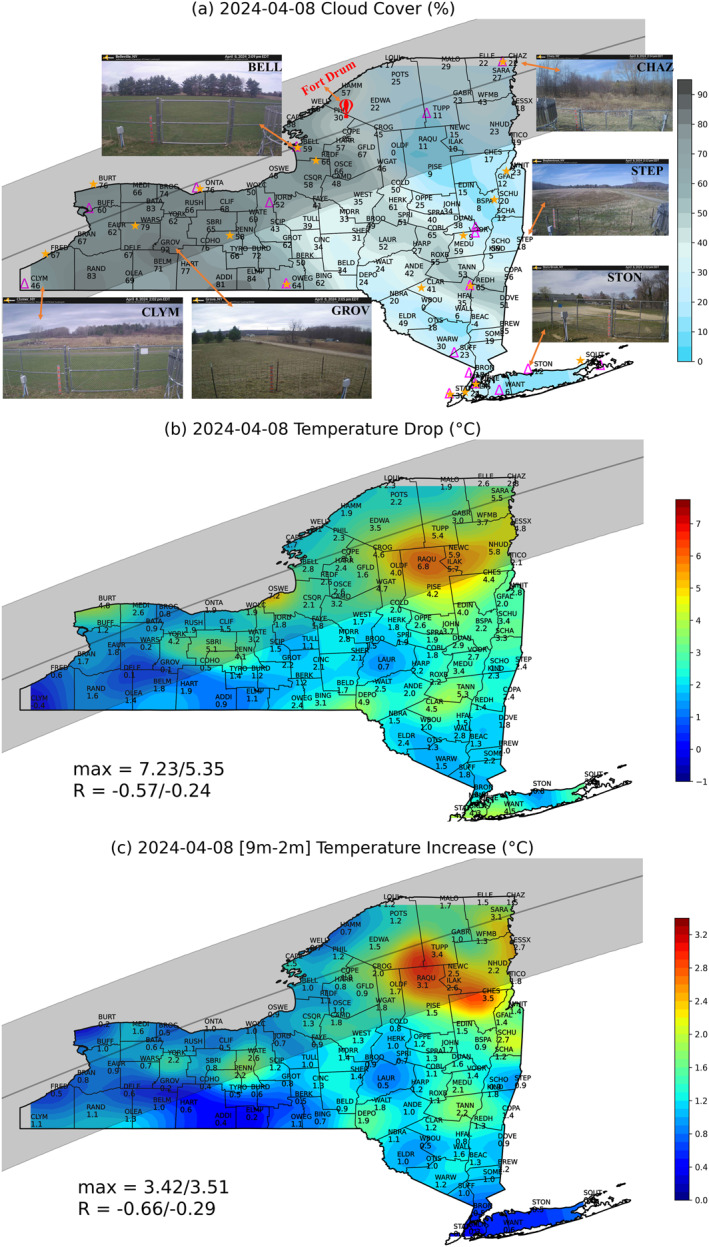
Maps (shading with station values) of the (a) estimated cloud cover (%) and the maximum changes (°C) in (b) T2m and (c) T9m–T2m. The maximum station value and the spatial correlation coefficient with the cloud cover for the stations on/off the totality path are shown in the legend. Camera images at ∼18 UTC at 6 sites are also shown in (a). The gray shaded area shows the totality path, and the gray line is the central line of totality. The magenta triangles and orange stars in (a) denote the profiler and flux sites, respectively. Fort Drum is also labeled in (a).

The analysis of the NYS Mesonet (NYSM) surface data collected during the 2017 partial eclipse demonstrated the value of the dense NYSM for documenting detailed responses of surface meteorological variables to the partial eclipse (Fowler et al., 2019). This study aims at conducting a comprehensive investigation on responses of the surface, PBL and land‐atmosphere interactions to the TSE on a regional scale using the unprecedented NYSM surface, profiler and flux data during the 2024 TSE (Figure 1a).

## 2 The 2024 Solar Eclipse Over New York State and NYSM Data

The 2024 TSE arrived at Jamestown in western NYS around 18 UTC (EDT+4) and exited from Plattsburgh, NY around 20:37 UTC (Figure 1a). The entire event lasted approximately 2.5 hr at any given location. The totality started shortly after 19:15 UTC in southwestern NY, ended just before 19:30 UTC in northeastern NY, and lasted for about 2–4 min depending on the location. There are five stages during a TSE. First contact (C1) is the first moment that the moon is visible against the sun, second contact (C2), totality (T) and the third contact (C3) are the beginning, peak and ending of totality, respectively, and fourth contact (C4) represents the end of the moon covering any part of the solar disk. For a partial eclipse, only C1, T (eclipse peak time) and C4 are valid.

The NYSM Standard Network consists of 126 sites across the state with an average spacing of ∼25 km (Figure 1a) (Brotzge et al., 2020). All stations collect 3–60 s raw measurements and 5‐min averages of standard meteorological variables including downward solar radiation (Srad) and have cameras to capture images every 5 min. Fifty‐five of the 126 sites were on the totality path (100% obscuration), and the rest experienced partial eclipses with at least 88% obscurations. In addition, the NYSM has two sub‐networks consisting of 17 profiler and 18 flux sites to provide continuous measurements of atmospheric vertical profiles and the surface energy budget, respectively (Covert, 2019; Shrestha et al., 2021). Each profiler site is equipped with a WindCube WLS‐100 scanning Doppler wind lidar (DWL) and a MP3000 A microwave radiometer (MWR) (Shrestha et al., 2021). The DWL provides 3D wind and aerosol profiles from 100 m to 7 km every 10–20 s; however, the data availability is usually limited within the PBL due to lack of aerosols above the PBL. The MWR provides retrieved temperature, humidity and liquid water profiles from surface to 10 km every 2 min but with less sensitivity and larger uncertainty above ∼4 km (Shrestha et al., 2021, 2022). The flux network measures both incoming and outgoing shortwave and longwave radiation, ground heat flux, 3D winds and carbon dioxide (CO_2_) concentration every 5 min and derives turbulent fluxes of momentum, sensible and latent heat every 30 min. All flux sensors are at 8 m above the ground level (AGL) except ground heat plates at 6 cm below the ground. There are seven profiler and eight flux sites on the totality path (Figure 1a). The NYSM has nine supersites where the standard, profiler and flux sites are closely collocated, and three of them, Ontario (ONTA), Belleville (BELL) and Chazy (CHAZ), are on the totality path (Figure 1a). The MWR temperature profiles are known to suffer from cold biases (Shrestha et al., 2022). An algorithm was developed to correct the MWR cold biases and is described in detail in Supporting Information S1.

To better capture the TSE on our site cameras, the sampling time was changed from the regular 5 min to 1 min. The TSE was clearly visible on the 1‐min camera images, and the camera automatically went to the night mode (black/white) during the totality. For example, at CHAZ (one of the best sites for viewing the totality due to clear sky) the camera showed dusk at 19:25 UTC and turned to the night mode during 19:26‐19:28 UTC (NYSM, 2024a). The camera images are also useful to validate sky and ground conditions as shown in Figure 1a and Section 3.3.

**Figure 2:**
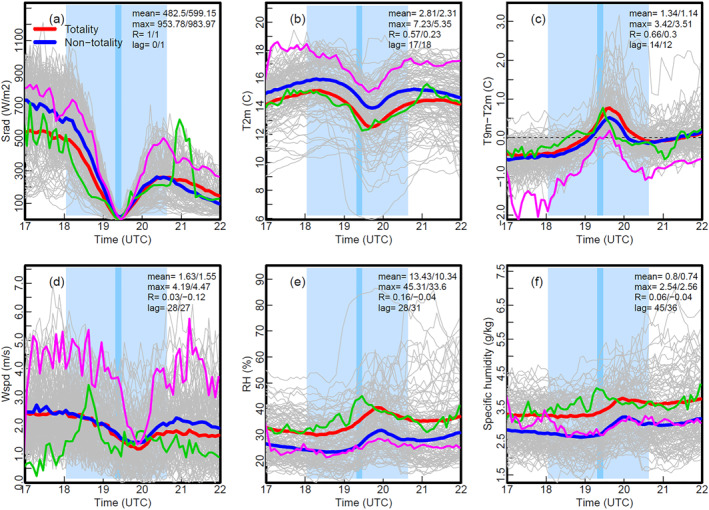
Time series of (a) downward solar radiation (W/m^2^), (b) T2m (°C), (c) T9m–T2m (°C), (d) 10m wind speed (m/s), (e) 2m relative humidity (%), and (f) 2m specific humidity (g/kg) from 17 to 22 UTC on 8 April 2024 from all 126 stations (gray lines). The thick red and blue lines show the averages of the stations on and off the totality, respectively. The light blue shaded area starts from the earliest C1 to latest C4 of the TSE for the 126 sites, and the dark blue area indicates the earliest to latest times of the peak totality. The mean and maximum (max) of the changes of the shown variables, the inter‐station correlation (R) between the solar radiation drop and the changes, and the mean time lag (in minutes, relative to T) for the stations on/off the totality path are given in the legends. The green and magenta lines show the values at BELL and CHAZ, respectively.

Statistics for total and partial eclipse sites were calculated separately (Figure 2). Similar to the method used in Fowler et al. (2019), minimum solar radiation, 2m temperature and wind speed, and maximum relative and specific humidity and near‐surface temperature inversion from the T to C4, and their departures from C1 were calculated. In addition, the time from T to the time when minimum (or maximum) values were recorded was calculated and referred to as the “time lag”. To better explain the observed surface responses, the cloud cover at C1 was estimated as (Srad_clear—Srad_measured)/Srad_clear, where Srad_measured and Srad_clear are the measured Srad and the clear sky value based on the model from Ineichen (2008).

## 3 Results

A snowstorm started on the night of 3 April and dumped over half a foot of snow overnight in the Adirondacks. The snow stayed on the ground until 7 April with a high‐pressure system and clear skies over the NYS, and the temperature rose above freezing and melted the snow. A low‐pressure system started to move across the NYS from the Ohio valley in the early afternoon on April 8 before the TSE. Middle and high clouds were visible on camera images and on the estimated‐cloud cover map (Figure 1a). During the TSE, the sky in the central Adirondacks was clear, providing the best eclipse views (Figure 1a). Two supersites (BELL and CHAZ) on the totality (Figure 1a) are selected to show the PBL and flux responses since BELL (CHAZ) represents cloudy (clear) conditions (Figure 1a).

### 3.1 Surface Responses

The NYSM 5‐min surface data at all 126 sites are analyzed for the 5‐hr period centered around the maximum eclipse (∼19:24 UTC) from 17 to 22 UTC. The Srad is directly impacted by the TSE, is inversely proportional to the obscuration of the sun during the TSE and is close to zero at the T (e.g., Founda et al., 2007; Kameda et al., 2009). At the C1, Srad ranges around 100–1037 Wm^−2^, indicating a mix of overcast and clear sky conditions (Figure 2a). The estimated cloud cover and camera images (Figure 1a) show overcasting in southwestern NYS and clear sky in the northern Adirondacks. As expected, Srad reaches its minimum values at the T, which is close to zero on the totality path and is well correlated with the obscuration for partial eclipse sites with a mean correlation coefficient of 0.88. Around the totality, Srad drops on average by 483 and 599 W m^−2^ on and off the totality path, respectively (Figure 2a). The larger Srad drop for the non‐totality stations is mainly due to relatively clear‐sky conditions at the downstate sites (Figure 1a). The Srad temporal and spatial variations are well captured by animated maps of 5 min Srad (NYSM, 2024b).

Air temperature at 2 m (T2m) AGL, temperature difference between 9 and 2 m AGL (T9m–T2m), 10 m wind speed, 2 m relative humidity (RH) and specific humidity all show consistent and significant responses to the total or partial eclipses at all 126 sites (Figures 2b–2f). Both the mean (2.8 vs. 2.3°C) and maximum (7.2 vs. 5.4°C) of the T2m drop are noticeably larger along the totality path (Figure 2b). Raquette Lake (RAQU) experienced the largest cooling (6.8°C) due to clear sky in the region (Figure 1b) and much calmer winds than neighboring stations (not shown). The maximum temperature drop of 7°C has been reported previously (Aplin et al., 2016; Kameda et al., 2009; Vogel et al., 2001), although Stewart and Rouse (1974) show 10°C drop at an Arctic station. Note that the 7.2°C drop at Oswego (OSWE) in Figure 1b is due to a sudden wind shift from southeasterly to northerly from Lake Ontario shore around 18:20 UTC, causing a ∼6°C temperature drop within 10 min. Cloud cover strongly affects the impact of the eclipse. This is confirmed by the significant spatial correlation between the T2m drop and cloud cover, which is −0.57 and −0.24 for the totality and non‐totality sites, respectively (Figures 1a and 1b). The T2m time lag is about 17 min on average (Figure 2b) and has been reported to be around 0–34 min in the literature (Kameda et al., 2009), which is attributed to the thermal inertia of the surface layer (Aplin & Harrison, 2003).

Under normal conditions, T2m is warmer than T9m during daytime; however, during the TSE, the surface cools faster through longwave radiation than the air, leading to a near‐surface temperature inversion of 0–2°C (Figure 2c). The T9m−T2m difference removes most of the wind change effect and thus represents the response to the Srad change better than T2m. This explains its stronger spatial correlation with cloud cover than the T2m drop (Figures 1b and 1c). Surface winds start to calm down from C1, reaching the minimum about 30 min after T and showing smaller differences between totality and non‐totality sites (Figure 2d). Both RH and specific humidity peaked about 30–40 min after T (Figures 2e and 2f), which is significantly later than the minimum of T2m. This suggests that the RH increase is not entirely due to the T2m drop, which decreases the saturation vapor pressure, but also due to changes in water vapor content that is affected by surface evaporation and upward mixing. Thus, there is a buildup in water vapor concentration near the surface during TSE, which contributes to the RH increase of ∼13% and ∼10% on and off the totality path, respectively (Figure 2e). The surface water vapor increase is likely due to reduced upward mixing since surface evaporation decreases as the surface cools. Prior studies have shown that the moisture in the surface layer can be diluted due to increased subsidence of dry air (Bhat & Jagannathan, 2012), and moistened (Mahmood et al., 2020) or no change (Turner et al., 2018) during TSEs. Such inconsistency is due to competing factors including surface cooling, reduced evaporation, reduced upward mixing, increased subsidence and so on and it deserves more investigation.

### 3.2 PBL Response

**Figure 3:**
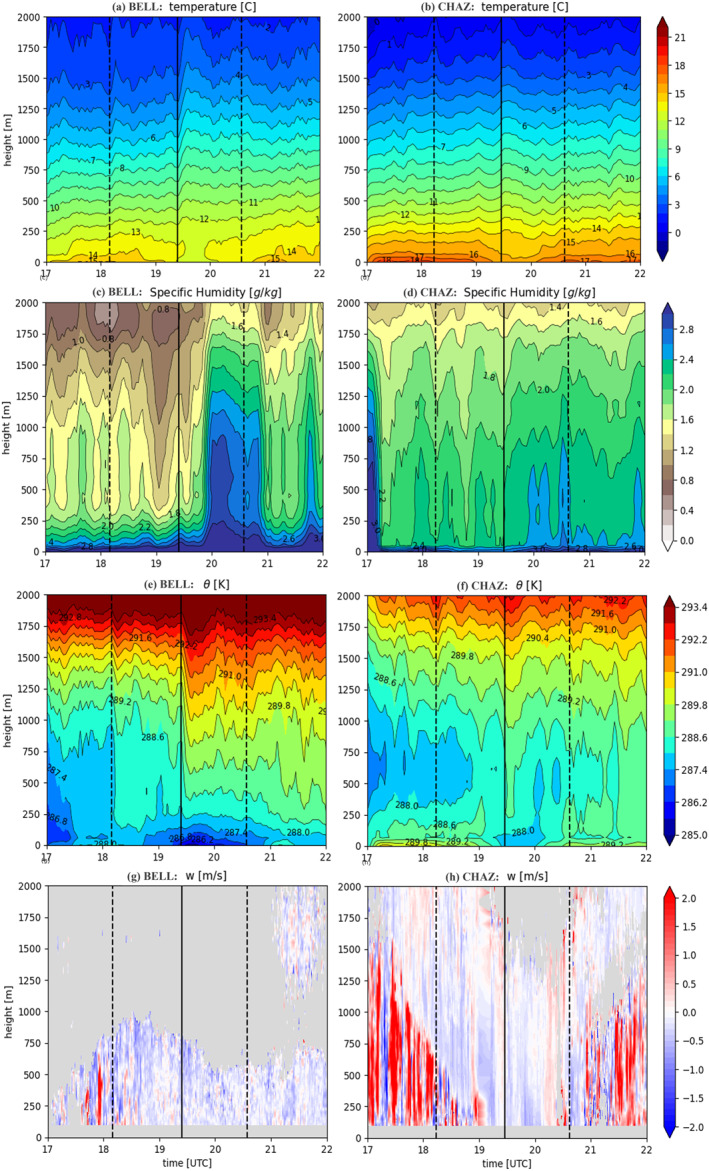
Profiles of (a–b) temperature (°C) (c–d) specific humidity (g/kg) (e–f) potential temperature (K) from MWR data, and (g–h) DWL vertical wind (m/s) at BELL (left panels) and CHAZ (right panels). The three vertical lines indicate the C1, T and C4 of the TSE.

The impact of the TSE expands beyond the surface into the PBL. Profiles of temperature, specific humidity, potential temperature and vertical wind speed are shown for BELL and CHAZ in Figure 3. The radiosonde data collected at Fort Drum (69 km Northeast of BELL) on April 7–8, 2024 (Figure 1a) as a part of Nationwide Eclipse Ballooning Project (NEBP) (Saad et al., 2024) are also shown in Figure S1 in Supporting Information S1 and compared against the MWR data at BELL. It is known that the MWR data have much coarser vertical resolution and large uncertainties in its humidity retrievals (e.g., Shrestha et al., 2022). They all can contribute to the discrepancies between MWR and radiosonde data discussed below. However, the MWR's high sampling rate (2 min) enables one to study rapid temporal changes, such as the responses to the TSE.

**Figure 4:**
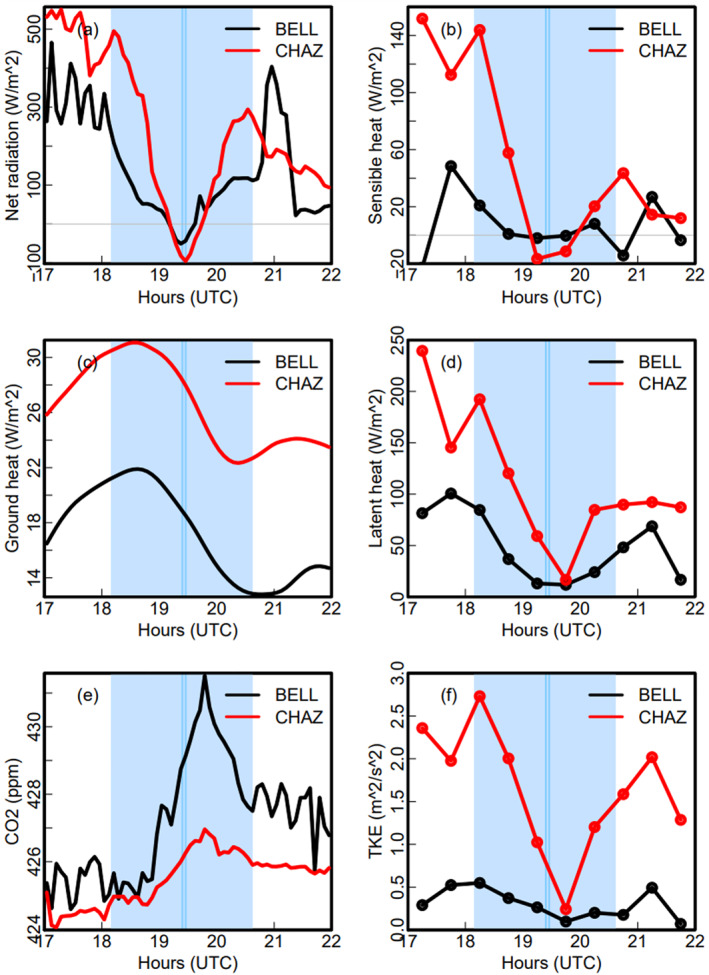
Time series of (a) net radiation, (b) sensible, (c) ground, and (d) latent heat fluxes, (e) CO_2_ concentration, (f) TKE at BELL (black) and CHAZ (red). The net radiation and ground heat fluxes are positive downward while the sensible and latent heat fluxes are positive upward. The light blue shaded area is from C1 to C4, and the dark blue lines for the Ts at the two sites.

The MWR data show that the eclipse‐induced cooling is largest at the surface and is confined below ∼200 m AGL (Figures 3a–3b, S1b–S1c in Supporting Information S1) in agreement with Mahmood et al. (2019). The radiosonde data show that the cooling extends up to ∼500 m in the PBL (Figure S1a in Supporting Information S1). Specific humidity clearly increases after the totality at both sites from the surface to as high as 2 km AGL at BELL, although CHAZ is much drier than BELL and the moistening is much smaller and shallower (Figures 3c and 3d, S1e–S1f in Supporting Information S1). The radiosonde specific humidity data show well‐defined PBL, reduced PBL height and systematic moistening within ∼500 m in the PBL after the TSE (Figure S1d in Supporting Information S1). Mahmood et al. (2020) found similar moistening using MWR data for the 2017 TSE in Kentucky, but it was confined in a much shallower layer (lowest 100 m). Such moistening is also consistent with the increase in water vapor mixing ratio in the surface layer associated with the evening transition on a normal day (Wingo & Knupp, 2015). Given the reduced latent heat flux during the TSE (Figure 4d), the increased specific humidity in the PBL has to result from reduced upward mixing of water vapor in the shallowed PBL, which is supported by the confinement of moistening in the PBL shown in the radiosonde data (Figure S1d in Supporting Information S1).

The MWR potential temperature (θ) profiles at BELL show a well‐mixed PBL of ∼1.2 km before the TSE, which is consistent with the radiosonde data, and then it started to collapse and transition to a shallow stable PBL with large positive dθ/dz right after the totality due to decay in heat and momentum fluxes and turbulent mixing associated with reduced surface heating, although the radiosonde data still show a well‐mixed layer after the totality, but much shallower (Figure 3e, S1g–S1h in Supporting Information S1). The PBL top at CHAZ before the TSE is slightly higher (∼1.5 km) than BELL likely because of stronger surface heating under clear sky and show smaller changes after the totality (Figure 3f, Figure S1i in Supporting Information S1). The DWL vertical winds switched from strong upward mixing before the totality to small downdrafts afterward (Figures 3g and 3h). This feature is much more pronounced at CHAZ than at BELL due to the clear sky. The shallowed PBL and stalled vertical mixing likely contribute to the moistening in the PBL after the totality shown in Figures 3c and 3d.

### 3.3 Surface Flux and CO_2_ Responses

Figure 4 shows the temporal evolution of surface net radiation, ground, sensible and latent heat fluxes, total kinetic energy (TKE) and CO_2_ concentration for BELL and CHAZ. Surface meteorological measurements at those two sites are shown in Figure 2. The clear sky at CHAZ results in much larger solar and net radiation drops than at BELL from C1 to T (Figures 2a and 4a). However, the two sites had the same surface cooling (2.8°C) (Figures 1b and 2b). This may be partly due to the larger drops in the upward latent and sensible heat fluxes at CHAZ, which offset the larger drops in downward radiation (Figures 4b and 4d). The sensible heat flux at CHAZ even changes to small negative values shortly before the totality (Figure 4b). The surface cooling also causes reductions (minimum near C4) in the downward ground heat flux (Figure 4c). The impacts of the TSE on surface energy fluxes shown in Figure 4 are expected due to the shut‐off of solar radiation and consistent with prior studies (e.g., Nymphas et al., 2012; Turner et al., 2018; Wood et al., 2019). The TSE calms the atmosphere down and weakens turbulence as shown by the TKE drop (Figure 4f), which is consistent with the reduced vertical motion (Figures 3g and 3h). The CO_2_ concentration starts to rise about an hour after C1 and reaches a maximum at ∼30 min after the totality (Figure 4e), as photosynthesis slows down with decreasing solar radiation. In addition, the reduced PBL depth and vertical mixing (Figure 3) result in the surface CO_2_ emissions not being mixed as deeply and quickly, contributing to the rise in near‐surface CO_2_ concentration. The CO_2_ response at BELL is much stronger than at CHAZ, which is likely due to weaker photosynthesis at CHAZ as evident by brown grass there (Figure 1a). Other 6 flux sites on the totality path show similar impacts on the surface fluxes and CO_2_ as BELL and CHAZ (not shown).

## 4 Summary

We analyzed surface data at the 126 NYSM sites on 8 April 2024 to quantify the impact of the TSE that passed through NYS. The eclipse started shortly after 18 UTC and ended at 20:37 UTC in NYS, with the totality lasting for ∼2–4 min at each site and occurring at ∼19:15–19:30 UTC. There are 55 NYSM stations on the totality path, while 71 stations have a partial eclipse with an obstruction of 88% or higher.

The NYSM data show a mean drop in Srad of 483 and 599 Wm^−2^ from C1 to T for the totality and non‐totality stations, respectively, as a result of more clouds on the totality path. The Srad decrease leads to a mean drop in T2m of 2.8°C (2.3°C) on (off) the totality path at ∼17 min after the totality. The cooling is limited to ∼200 m AGL into the atmosphere in the MWR data but extends up to ∼500 m AGL in the radiosonde data. The cooling calms down vertical motion and turbulence mixing, which leads to the collapse of the PBL and an accumulation of water vapor near the surface despite a large drop in surface evaporation during the totality. Combined with the T2m drop, this leads to a large mean increase of 10%–13% in surface RH and 0.74–0.8 g/kg in surface specific humidity (29%) after the totality. The moistening also expands to the PBL likely due to reduced vertical mixing in a shallowed PBL. Another robust response is in the temperature vertical gradient (T9m–T2m), which turns from negative before the totality into positive (i.e., inversion) shortly afterward. Surface sensible, latent and ground heat fluxes all decrease during the TSE. We also found a robust rise in near‐surface CO_2_ concentration with a peak 30 min after the totality, likely due to the shutdown of photosynthesis and reduced vertical mixing.

The NYSM's dense surface network and co‐located surface, profiler, and flux sites enable us to characterize the responses of the surface meteorological and flux variables and the PBL to the TSE and partially explain observed responses. This unprecedented NYSM data set for the rare 2024 TSE has more values than what is described in this letter. It can be used to conduct in‐depth study on the TSE's impacts, to understand observed responses, and to validate weather forecasting models and reanalysis products in simulating the TSE's impacts. There are other data available, such as radiosonde data at Brockport and Oswego besides Fort Drum from NEBP (Saad et al., 2024) and other profiling data collected during a NYSM‐led field campaign in April at three NYSM sites. They can be used to validate the results shown in this study and integrated with the NYSM data to provide the community a more comprehensive data set.

## Conflict of Interest

The authors declare no conflicts of interest relevant to this study.

## Supporting information

Supporting Information S1

## Data Availability

The NYSM data and camera images are available for requesting at https://www.nysmesonet.org/weather/requestdata according to the NYSM data policy stated on the webpage.
