# Supplementary material for: Characterizing the Impacts of 2024 Total Solar Eclipse Using New York State Mesonet Data
Source: Geophys Res Lett. 2024 Nov 20;51(22):e2024GL112684. doi: 10.1029/2024GL112684 (PMC11578864; doi:10.1029/2024GL112684)
Supplement: Supplementary file 1 — Supporting Information S1 [file GRL-51-0-s001.docx]

*Geophysical Research Letters*

Supporting Information for

**Characterizing the Impacts of 2024 Total Solar Eclipse Using New York State Mesonet Data**

Junhong Wang^1^, Aiguo Dai^2^, Chau-Lam Yu^1^, Bhupal Shrestha^1^, D. J. McGuinnes^1^, and Nathan Bain^1^

^1^New York State Mesonet, SUNY University at Albany, Albany, NY, USA

^2^Department of Atmospheric and Environmental Sciences, SUNY University at Albany, Albany, NY, USA

**Contents of this file**

Text S1

Figures S1 to S2

**Introduction**

In this supporting document, we describe the bias correction (BC) scheme developed recently to correct the cold bias in the MWR temperature data. Using High-Resolution Rapid Refresh (HRRR) analysis as a reference, we first compute and collect the observational error in the retrieved temperature profile at each MWR site at 00, 06, 12, and 18 UTC from March 13th to May 31st. Meanwhile, principal component analysis (PCA) is used to extract six leading principal components of the 35 brightness temperature (Tb) channels during this two-and-half month period, which contain the essential signals of observed Tb. Multiple linear regression is then used to regress the collected observational errors onto the six leading principal components to obtain the BC coefficients at each level. With the BC coefficients, the derived BC scheme uses the observed Tbs at each site as input variables to predict the corresponding bias at each level of the retrieved temperature profiles.

Text S1.

Figure S1 shows the temperature, potential temperature, and specific humidity profiles at five times before the TSE (17:51 UTC), before the totality (18:27 and 19:00 UTC), after the totality (20:00 UTC) and after the TSE (21:02 UTC) from Fort Drum radiosonde data and MWR data at BELL and CHAZ. Fort Drum is 69 km Northeast of BELL (Fig. 1a). The temperature correction is positive above 1 km at both BELL and CHAZ, with a magnitude of about 5 K near 2 km (Fig. S1b and S1c). The bias-corrected temperature (Fig. S1b) and potential temperature (Fig. S1h) profiles at BELL show improved agreement with the radiosonde profiles at Fort Drum (Fig. S1a, g).

Using 31 radiosonde profiles collected at Fort Drum on April 7th and 8th as references, Figure S2 compares the MWR’s mean error and standard deviation of the temperature, potential temperature, and specific humidity retrievals at BELL before and after the bias correction. It is clear that the BC scheme effectively removes the substantial cold bias in the temperature and potential temperature above 1 km, consistent with the improved agreement shown in Fig. S1. Because the BC scheme only corrects the temperature, the impact of the correction on the retrieved specific humidity is minimal.


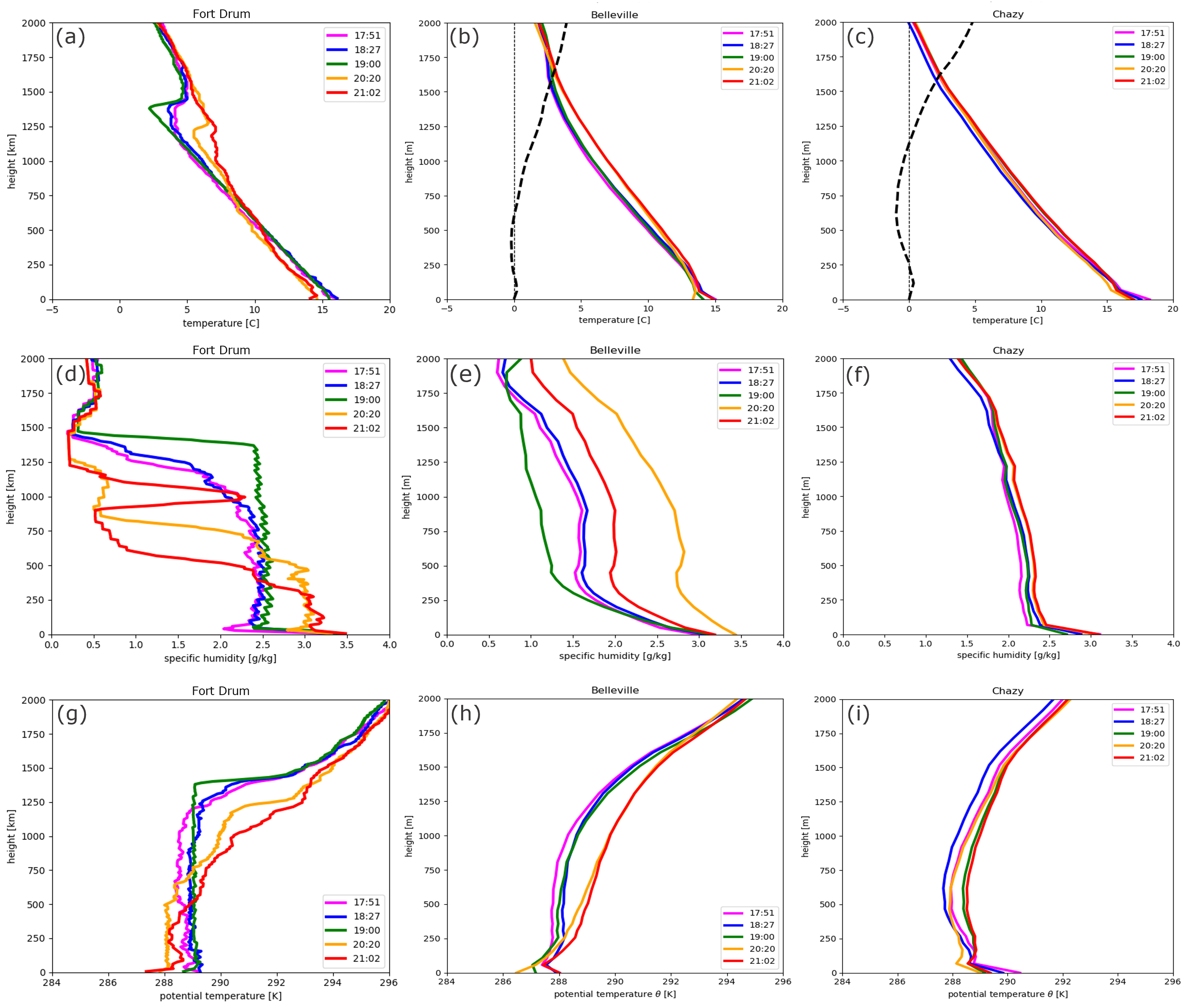


Figure S1. Temperature profiles below 2 km at five times from a) Fort Drum radiosonde data, b) MWR data at BELL, and c) MWR data at CHAZ. The Black dashed line in (b) and (c) shows the

profiles of temperature bias correction. The middle (d, e, f) and bottom rows (g, h, i) are similar to the top row but for specific humidity and potential temperature, respectively.


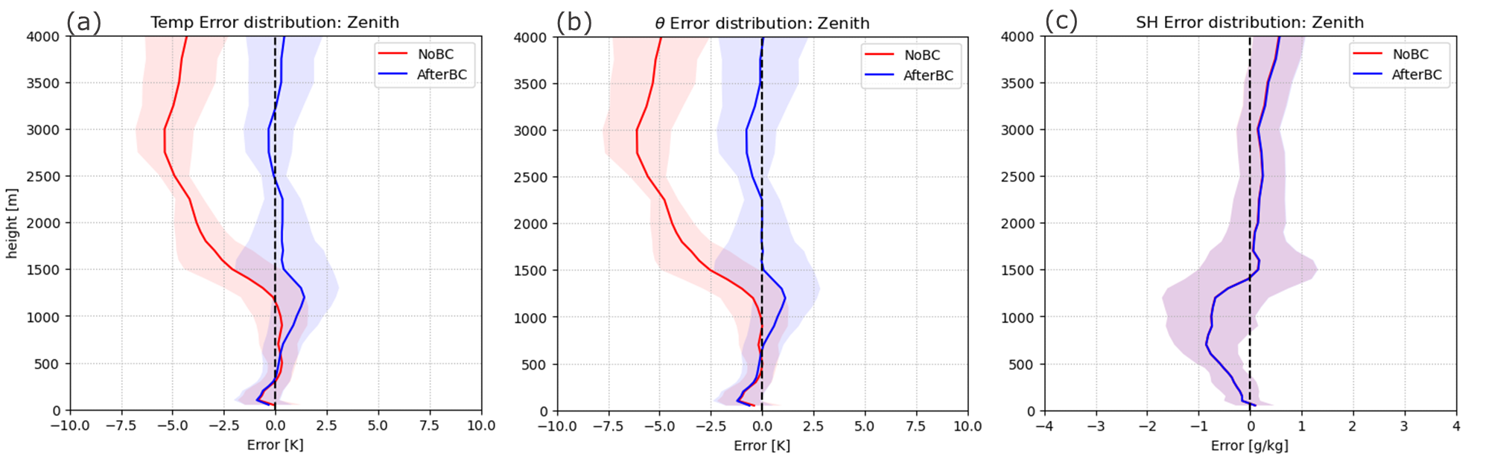


Figure S2. (a) Profiles of mean error (solid line) and standard deviation (shaded) of MWR temperature retrieval at BELL before (red) and after (blue) the bias correction. Fort Drum radiosonde soundings launched on April 7th and 8th are used as verification reference. Panels (b) and (c) are similar to (a), but for potential temperature and specific humidity, respectively.
